# Complete plastid genome and phylogenetic analysis of *Hypericum wightianum* (Hypericaceae)

**DOI:** 10.1080/23802359.2026.2699496

**Published:** 2026-07-09

**Authors:** Dan Wu, Rouhui Zhao, Anqi Yu, Xiaojuan Li, Yuchen Yan, Geng Gao, Xinyi Pan, Shan Jiang, Jianke Yang

**Affiliations:** ^a^ School of Clinical Medicine, Wannan Medical University, Wuhu, China; ^b^ School of Medical Imageology, Wannan Medical University, Wuhu, China; ^c^ School of Pharmacy, Wannan Medical University, Wuhu, China; ^d^ School of Basic Medical Sciences, Wannan Medical University, Wuhu, China

**Keywords:** *Hypericum*, plastome, *Monanthema*, phylogeny

## Abstract

*Hypericum wightianum* is a medicinal plant (section *Monanthema)* . In this study, we assembled, annotated and characterized the complete plastid genome of *H. wightianum*. The plastome is 137,760 bp in length and exhibits a standard quadripartite layout. In total, 119 genes were identified, comprising 35 tRNAs, 8 rRNAs, 3 pseudogenes, and 73 protein-coding genes with biased synonymous codon usage. Phylogenetic inference indicated that *H. wightianum* and *H. petiolulatum* form a well-supported sister clade, suggesting a close evolutionary relationship between sections *Monanthema* and *Elodeoida*. This data provide critical resources for species delimitation and evolutionary studies within the genus *Hypericum*.

## 1. Introduction

*Hypericum* includes exceeding 400 species classified into 36 sections (Robson 2012; Borsch et al. 2020). High interspecific morphological similarity and intraspecific phenotypic plasticity complicate traditional taxonomic resolution of the genus (Crockett and Robson 2011; Liu et al., 2023). *Hypericum wightianum* Wall. ex Wight & Arn. 1934 (sect. *Monanthema*), an annual herb in southern and southeastern Asia, is pharmacologically significant due to its richness in bioactive compounds, including phenols, flavonoids, glycosides, and alkaloids (Robson 2012; Balaperiasamy et al. 2013; Fu et al. 2025). Nevertheless, *H. wightianum* is frequently misidentified when relying solely on traditional phenotypic traits, highlighting an urgent need for robust molecular markers.

The plastid genome (plastome) typically exists as a circular molecule with a characteristic quadripartite structure, ranging from 120 to 180 kb. The plastome serves as an invaluable molecular resource, widely utilized for phylogenetic inference and species delimitation (Xiao-Ming et al. 2017; Cauz‐Santos 2025). Despite this, relatively few Hypericaceae plastomes have been released in public databases because of their structural complexity and assembly challenges (Claude et al. 2025; Yan et al. 2025). In particular, the full plastome of *H. wightianum* currently remains undocumented.

Here, we reconstructed the complete *H. wightianum* plastome by integrating second-generation and PacBio HiFi sequencing data. Comparative and phylogenetic analyses resolved its position within *Hypericum*. These findings facilitate the identification, conservation, and evolutionary study of this medicinal plant.

## 2. Materials and methods

### 2.1. Sampling

**Figure 1.**
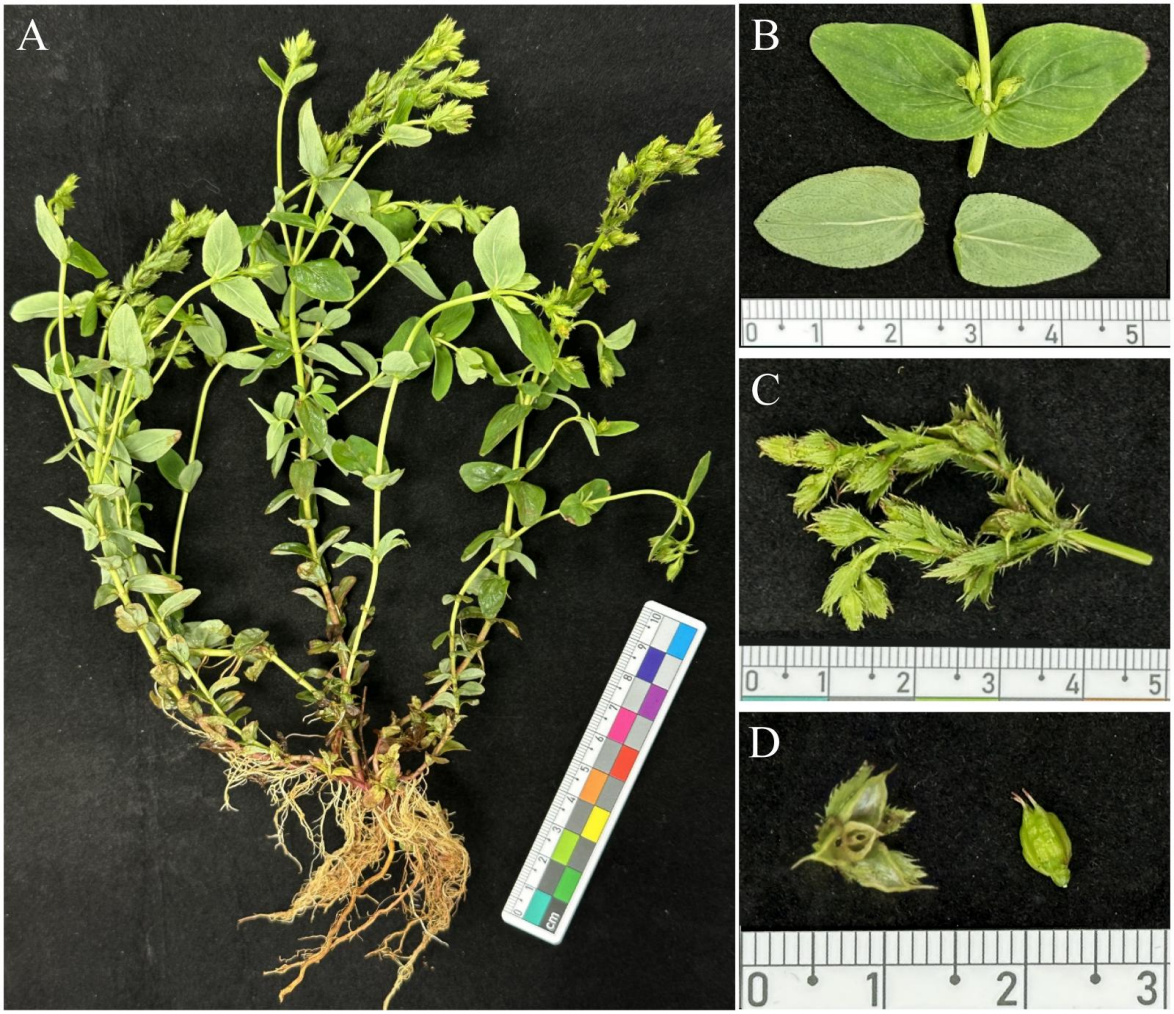
Morphological features of *H. wightianum*. (A) Whole plant, showing the herbaceous habit with tufted stems erect to decumbent from a creeping base. (B) Sessile leaf with pale laminar glands and black intramarginal glands. (C) Dichasial cyme; sepals with marginal, stalked black glandular teeth. (D) Subglobose capsule. Representative images were acquired by G. Gao and the corresponding author in-house.

*H. wightianum* was collected in Kunming, Yunnan Province, China (25°16′18.78″ N, 102°49′58.84″ E). After taxonomic verification, samples were rapidly frozen and preserved at −80 °C. A voucher (YHW230801) was deposited in the Herbarium of Chinese Medicinal Herb at Wannan Medical College (HCMH, contact: Jianke yang, Email: ajiankebc@wnmc.edu.cn) (Figure 1).

### 2.2. DNA extraction and sequencing

Genomic DNA was isolated following the optimized CTAB protocol (Doyle and Doyle 1987). Short-read sequencing was performed on the DNBSEQ-T7 platforms (150-bp paired-end) using 300-bp inserts libraries. Simultaneously, PacBio HiFi libraries were sequenced on the Revio system and raw data were subsequently processed to yield HiFi reads (N50 ≈ 16 kb).

### 2.3. Plastome assembly and annotation

The plastome was de novo assembled utilizing GetOrganelle v1.7.3 (Jin et al. 2020). And long HiFi reads resolved ambiguous nodes, resulting in two circular isomers. Annotation was performed by GeSeq (Tillich et al. 2017) and refined through manual corrections. Finally, the complete plastome was visualized with CPGView (Liu et al., 2023).

### 2.4. Phylogenetic analysis

Total 31 Hypericaceae plastomes were retrieved from NCBI, with *Mesua ferrea* and *Kielmeyera appariciana* as outgroups. Sequences were aligned *via* MAFFT v.7.515 (Katoh and Standley 2013) and curated in Geneious (Kearse et al. 2012). Phylogenetic trees were reconstructed using Maximum Likelihood (RAxML GUI 2.0; 1,000 bootstrap replicates (Edler et al. 2021)) and Bayesian Inference (MrBayes v.3.2.7a; 10 million generations, 25% burn-in (Ronquist et al. 2012)). Alignment-free phylogeny was also inferred *via* JolyTree based on the Balanced Minimum Evolution (BME) method (Criscuolo 2019; Yang et al. 2024). Final trees were visualized and annotated in Evolview v.3.0 (Subramanian et al. 2019).

## 3. Results

**Figure 2.**
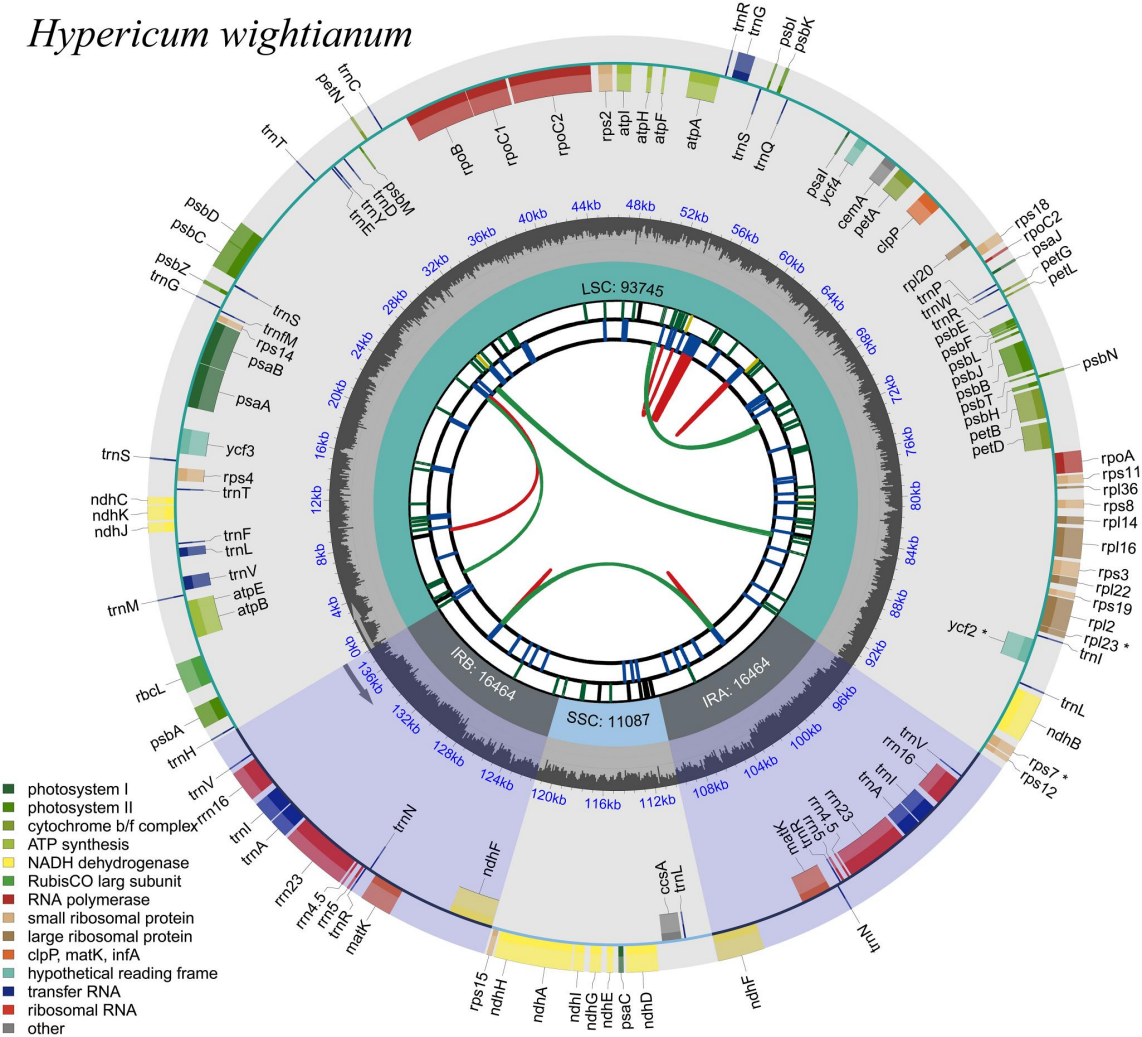
Schematic diagram of *H. wightianum* plastome. Concentric tracks (from outside to inside) indicate: (1) Gene arrangement, where different colors denote functional categories. Pseudogenes are labled by asterisks (*). arrows indicate the direction of transcription. (2) GC content, represented by the dark gray shading. (3) Lengths and boundaries of the four plastome regions: Large single-copy (LSC), small single-copy (SSC), and inverted repeats (IRA/IRB). (4–6) distribution of microsatellites (SSRs), tandem repeats, and dispersed repeats, respectively.

The circular *H. wightianum* plastome (137,760 bp; 37.1% GC) was assembled with an average sequencing depth of 37,431 x (Supplementary Figure S1). It displays a standard quadripartite layout: a large single-copy (LSC; 93,745 bp), a small single-copy (SSC; 11,087 bp), and a pair of inverted repeats (IRa/IRb; 16,464 bp each) (Figure 2). Strikingly, the IRs show marked contraction, encoding only 11 genes-a substantial reduction in both length and gene content compared to typical Hypericaceae plastomes. Annotation yielded 119 genes, comprising 73 protein-coding genes (PCGs), 35 tRNAs, 8 rRNAs, and 3 pseudogenes (*ycf2*, *rps7* and *rpl23*) carrying internal stop codons. Introns were also identified in eight PCGs and seven tRNAs (Supplementary Figure S2). Remarkably, *trnK-UUU* was not observed, suggesting a potential gene loss event in this lineage.

Additionally, we evaluated the relative synonymous codon usage (RSCU) patterns for the *H. wightianum* plastome. The results revealed eight codons displayed strong preference (RSCU > 1.6, max: TTA, 2.12) and 21 codons were significantly underrepresented (RSCU < 0.6; min: CTG, 0.32) (Supplementary Table S1). Intriguingly, despite the absence of *trnK-UUU* gene, the RSCU value for the corresponding AAA codon (Lysine) remained relatively high at 1.55.

**Figure 3.**
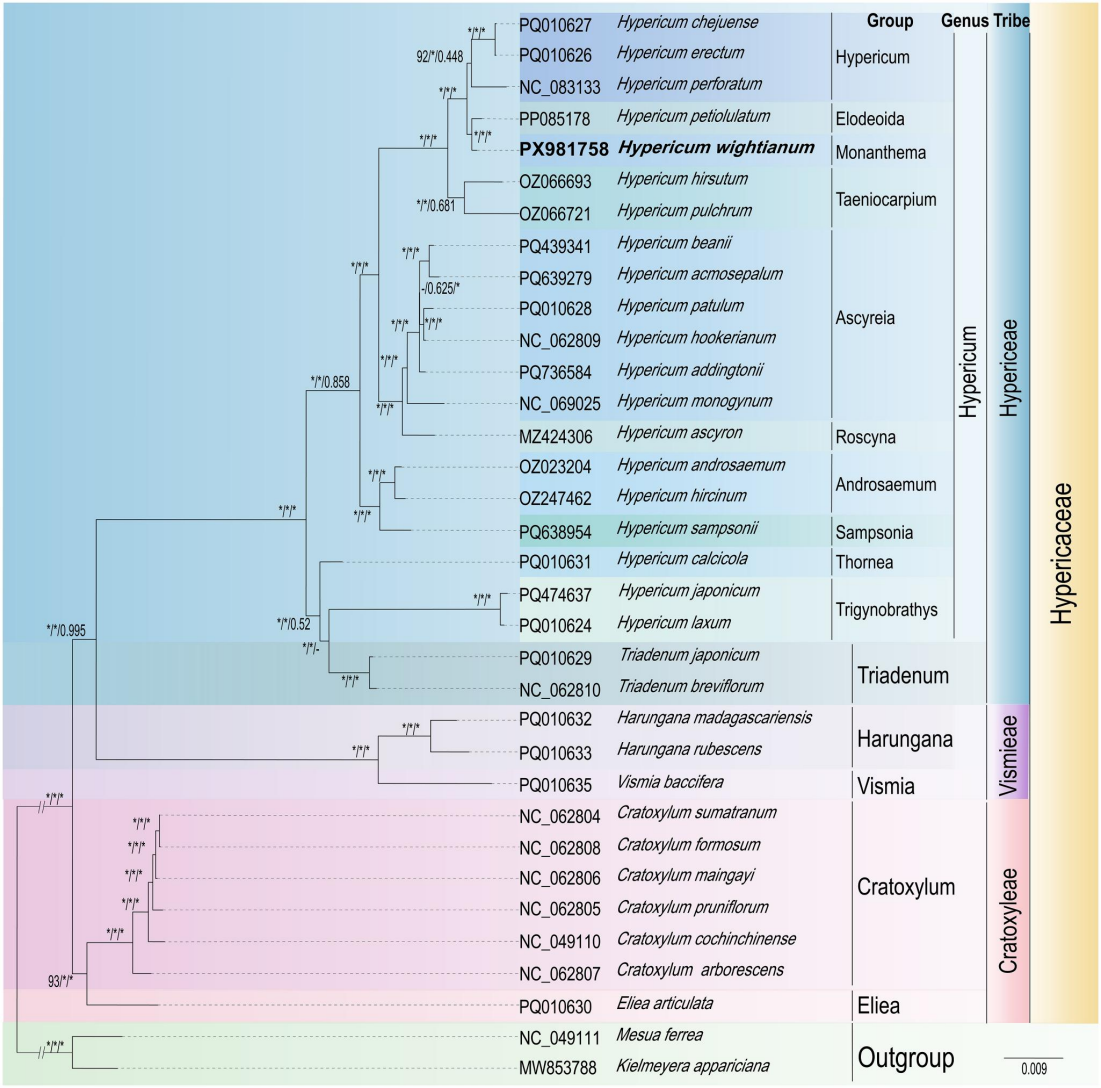
Phylogenetic tree of 32 hypericaceae species and two outgroups. The tree was reconstructed using maximum likelihood (ML), Bayesian inference (BI) and balanced minimum evolution (BME) methods. Species names in bold black font indicate plastome newly assembled in this study. ML bootstrap (BS) values, BI posterior probabilities (PP), and BME confidence levels (CL) are displayed at the nodes (BS/PP/CL). Asterisks (*) denotes high support (BS/CL > 90, PP > 0.9). GenBank accessions for the plastomes curated in this study are listed as follows: PQ010627 (Claude et al. 2025), PQ010626 (Claude et al. 2025), NC_083133 (Liu et al., 2023), PP085178 (Zou et al. 2024), PX981758 (this study), OZ066693 (unpublished), OZ066721 (unpublished), PQ439341 (Yan et al. 2025), PQ639279 (Yan et al. 2025), PQ010628 (Claude et al. 2025), NC_062809 (Sudmoon et al. 2022), PQ736584 (Yan et al. 2025), NC_069025 (unpublished), MZ424306 (Claude et al. 2022), OZ023204 (unpublished), OZ247462 (unpublished), PQ638954 (unpublished), PQ010631 (Claude et al. 2025), PQ474637 (Claude et al. 2025), PQ010624 (Claude et al. 2025), PQ010629 (Claude et al. 2025), NC_062810 (Sudmoon et al. 2022), PQ010632 (Claude et al. 2025), PQ010633 (Claude et al. 2025), PQ010635 (Claude et al. 2025), NC_062804 (Sudmoon et al. 2022), NC_062808 (Sudmoon et al. 2022), NC_062806 (Sudmoon et al. 2022), NC_062805 (Sudmoon et al. 2022), NC_049110 (Jin et al. 2020), NC_062807 (Sudmoon et al. 2022), PQ010630 (unpublished), NC_049111 (Jin et al. 2020), and MW853788 (Trad et al. 2021).

Phylogenetic trees (ML, BI, and BME) for Hypericaceae produced highly consistent topologies, with only slightly variations in support values. Most nodes received strongly support, with Bootstrap (BS) values > 90% and Posterior Probabilities (PP) > 0.90 (Figure 3). The 32 Hypericaceae species were partitioned into three distinct lineages: (I) *Hypericeae*, (II) *Vismieae*, and (III) *Cratoxyleae*. The *Hypericum* did not form a monophyletic group; instead, certain *Hypericum* taxa clustered within *Triadenum*. Additionally, *H. wightianum* and *H. petiolulatum* formed a sister clade that clustered with species of sect. *Hypericum*, suggesting a close phylogenetic affinity among sections *Hypericum*, *Elodeoida*, and *Monanthema.*

## 4. Discussion

While maintaining high structural synteny with other Hypericaceae species, the assembled *H. wightianum* plastome exhibits a marked length reduction, driven primarily by substantial contraction of IR regions. This phenomenon may represent a significant evolutionary trait for this species (Claude et al. 2025). Unlike most angiosperms where *trnK-UUU* is conserved, *H. wightianum* plastome shows complete *trnK-UUU* attrition and *matK* translocation to the IR. This shift appears linked to the evolutionary decoupling of *matK* from its host intron (Duffy et al. 2009; Claude et al. 2022). Crucially, the relocation of *matK* results in a two-copy state, potentially increasing gene dosage to facilitate the splicing efficiency of Group II introns (Barthet and Hilu 2007). Beyond structural changes, its 73 PCGs exhibit a distinct codon usage bias. This preference may be the result of the combined effects of natural selection, neutral mutations, and genetic drift (Yang et al. 2021; Ding et al. 2023). Additionally, the AAA (Lysine) codon maintains a high RSCU (1.55) even in the absence of *trnK-UUU*, suggesting functional compensation potentially driven by wobble pairing or the import of nuclear-encoded tRNA.

Our phylogenetic inference across three methods generated a robust and congruent topology, confirming the value of plastome in resolving the evolutionary relationships within Hypericaceae (Liu et al., 2023). The phylogeny indicated that *H. wightianum* (sect. *Monanthema*) clustered with *H. petiolulatum* (sect. *Elodeoida*), a close affinity supported by shared traits such as herbaceous habit and black glandular dots (Robson 2012). Consistent with previous studies, we corroborated the monophyly of three Hypericaceae tribes with Cratoxyleae as basal (Claude et al. 2025). Furthermore, *Hypericum* was recovered as non-monophyletic (nesting *Triadenum* species). This suggests that the current generic circumscriptions in Hypericaceae may benefit from further refinement based on more extensive molecular and morphological evidence.

## Supplementary Material

Figure S2.pdf

Supplementary Table S1.docx

Figure S1.pdf

## Data Availability

The plastome sequence are openly available in GenBank of NCBI (https://www.ncbi.nlm.nih.gov/) under the accession no. PX981758. Relevant BioProject, BioSample, and SRA numbers are PRJNA1480253, SAMN61021137, and SRR39242342, respectively.
