# Supplementary figures and images for: Complete plastid genome and phylogenetic analysis of Hypericum wightianum (Hypericaceae)
Source: Mitochondrial DNA B Resour. 2026 Jul 9;11(8):925–9. doi: 10.1080/23802359.2026.2699496 (PMC13353384; doi:10.1080/23802359.2026.2699496)

A

## Cis-splicing Genes

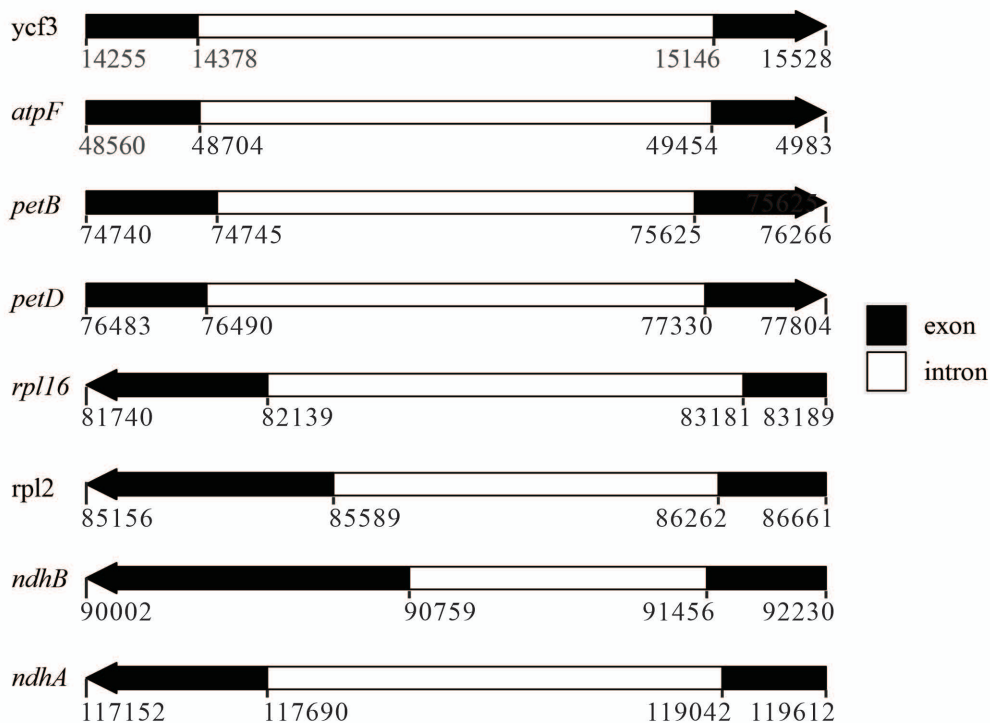

B

## Trans-splicing Genes

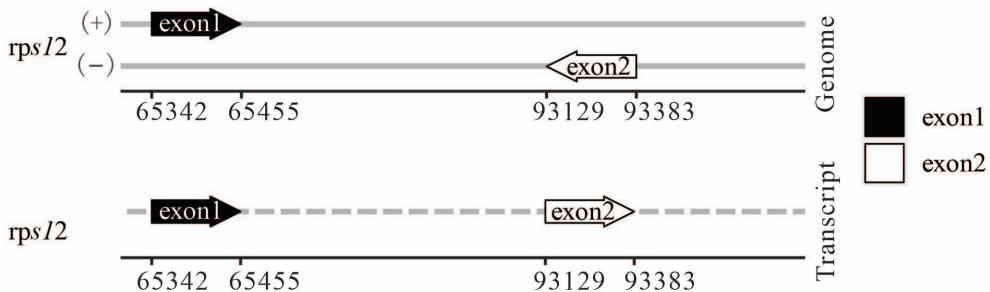

Supplement: Figure S2.pdf [file TMDN_A_2699496_SM9154.pdf]

# Depth Coverage along Sequence

Four continuous regions (total length: 137760 bp)

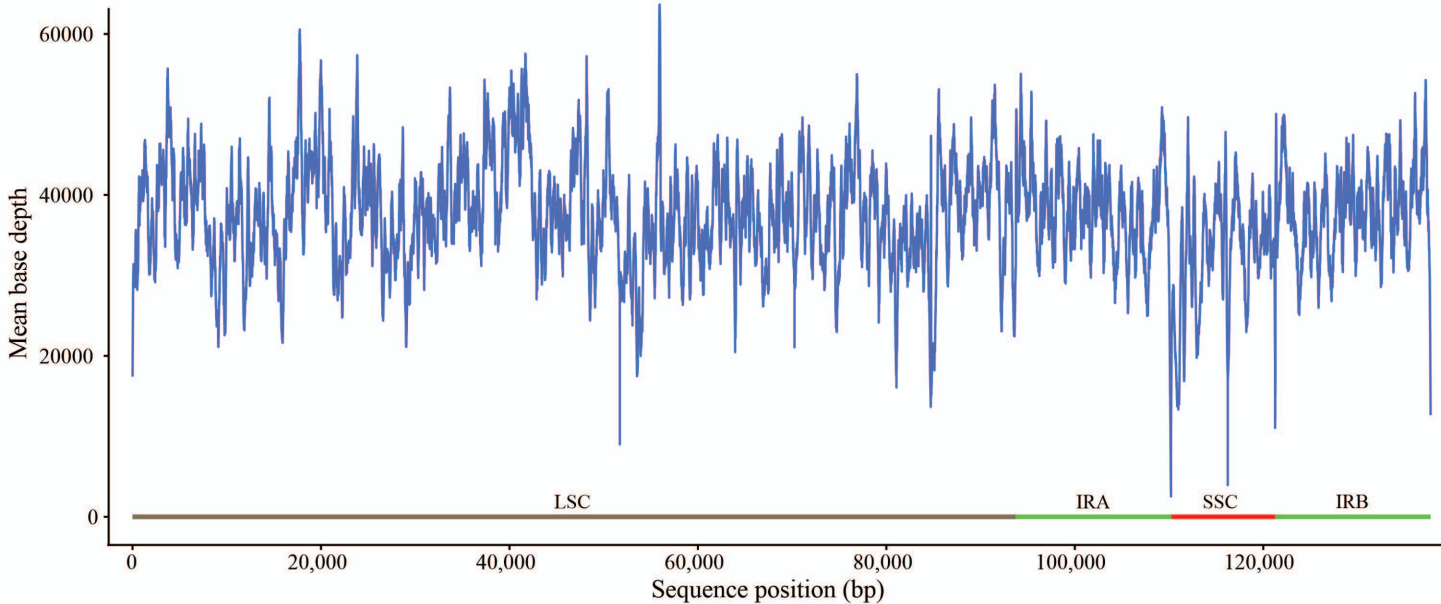

Supplement: Figure S1.pdf [file TMDN_A_2699496_SM9152.pdf]
