# Supplementary material for: Complete plastid genome and phylogenetic analysis of Hypericum wightianum (Hypericaceae)
Source: Mitochondrial DNA B Resour. 2026 Jul 9;11(8):925–9. doi: 10.1080/23802359.2026.2699496 (PMC13353384; doi:10.1080/23802359.2026.2699496)
Supplement: Supplementary Table S1.docx [file TMDN_A_2699496_SM9153.docx]

**Table S1.** Overall relative synonymous codon usage (RSCU) values from 73 protein-coding genes (CDSs) of *H. wightianum* plastome. Codons with RSCU values > 1.6 were defined as over-represented, while those with values < 0.6 were classified as under-represented.

| AA | Codon | Number^b^ | RSCU^a^ | AA | Codon | Number^b^ | RSCU^a^ |
| --- | --- | --- | --- | --- | --- | --- | --- |
| Ala | GCT** | 531 | 1.79 | Leu | TTA** | 763 | 2.12 |
|  | GCC | 194 | 0.65 |  | TTG | 383 | 1.07 |
|  | GCA | 333 | 1.12 |  | CTT | 463 | 1.29 |
|  | GCG* | 132 | 0.44 |  | CTC* | 140 | 0.39 |
| Arg | AGA** | 286 | 1.65 |  | CTA | 293 | 0.82 |
|  | AGG* | 102 | 0.59 |  | CTG* | 113 | 0.32 |
|  | CGA | 251 | 1.44 | Lys | AAA | 749 | 1.55 |
|  | CGC* | 91 | 0.52 |  | AAG* | 220 | 0.45 |
|  | CGG* | 85 | 0.49 | Phe | TTT | 874 | 1.45 |
|  | CGT | 228 | 1.31 |  | TTC* | 332 | 0.55 |
| Asn | AAC* | 167 | 0.42 | Pro | CCT** | 334 | 1.61 |
|  | AAT | 624 | 1.58 |  | CCC | 166 | 0.80 |
| Asp | GAT | 536 | 1.51 |  | CCA | 228 | 1.10 |
|  | GAC* | 172 | 0.49 |  | CCG* | 103 | 0.50 |
| Cys | TGT | 164 | 1.55 | Ser | TCT** | 389 | 1.71 |
|  | TGC* | 48 | 0.45 |  | TCC | 194 | 0.85 |
| Gln | CAA | 541 | 1.59 |  | TCA | 238 | 1.05 |
|  | CAG* | 139 | 0.41 |  | TCG | 137 | 0.60 |
| Glu | GAA | 725 | 1.52 |  | AGC* | 106 | 0.47 |
|  | GAG* | 231 | 0.48 |  | AGT | 299 | 1.32 |
| Gly | GGA | 540 | 1.55 | Thr | ACT** | 424 | 1.68 |
|  | GGC* | 165 | 0.47 |  | ACC | 163 | 0.65 |
|  | GGG | 221 | 0.63 |  | ACA | 302 | 1.20 |
|  | GGT | 470 | 1.35 |  | ACG | 119 | 0.47 |
| His | CAC* | 102 | 0.45 | Tyr | TAT** | 571 | 1.64 |
|  | CAT | 355 | 1.55 |  | TAC* | 125 | 0.36 |
| Ile | ATT | 870 | 1.57 | Val | GTT | 420 | 1.56 |
|  | ATC* | 295 | 0.53 |  | GTC* | 154 | 0.57 |
|  | ATA | 495 | 0.90 |  | GTA | 382 | 1.42 |
| Stop | TAA** | 39 | 1.60 |  | GTG* | 120 | 0.45 |
|  | TAG | 18 | 0.74 | Trp | TGG | 342 | 1.00 |
|  | TGA | 16 | 0.66 | Met | ATG | 447 | 1.00 |

^a^ overall values of RSCU, ^b^ total number of codons, AA: amino acid, * RSCU < 0.6, ** RSCU > 1.6.
